# Identification of Unprecedented Anticancer Properties of High Molecular Weight Biomacromolecular Complex Containing Bovine Lactoferrin (HMW-bLf)

**DOI:** 10.1371/journal.pone.0106568

**Published:** 2014-09-15

**Authors:** Fawzi Ebrahim, Jayanth Suryanarayanan Shankaranarayanan, Jagat R. Kanwar, Sneha Gurudevan, Uma Maheswari Krishnan, Rupinder K. Kanwar

**Affiliations:** 1 Nanomedicine-Laboratory of Immunology and Molecular Biomedical Research, School of Medicine, Faculty of Health, Deakin University, Geelong, Victoria, Australia; 2 Centre for Nanotechnology & Advanced Biomaterials (CeNTAB), School of Chemical & Biotechnology, SASTRA University, Thanjavur, India; National Research Council of Italy, Italy

## Abstract

With the successful clinical trials, multifunctional glycoprotein bovine lactoferrin is gaining attention as a safe nutraceutical and biologic drug targeting cancer, chronic-inflammatory, viral and microbial diseases. Interestingly, recent findings that human lactoferrin oligomerizes under simulated physiological conditions signify the possible role of oligomerization in the multifunctional activities of lactoferrin molecule during infections and in disease targeting signaling pathways. Here we report the purification and physicochemical characterization of high molecular weight biomacromolecular complex containing bovine lactoferrin (≥250 kDa), from bovine colostrum, a naturally enriched source of lactoferrin. It showed structural similarities to native monomeric iron free (Apo) lactoferrin (∼78–80 kDa), retained anti-bovine lactoferrin antibody specific binding and displayed potential receptor binding properties when tested for cellular internalization. It further displayed higher thermal stability and better resistance to gut enzyme digestion than native bLf monomer. High molecular weight bovine lactoferrin was functionally bioactive and inhibited significantly the cell proliferation (p<0.01) of human breast and colon carcinoma derived cells. It induced significantly higher cancer cell death (apoptosis) and cytotoxicity in a dose-dependent manner in cancer cells than the normal intestinal cells. Upon cellular internalization, it led to the up-regulation of caspase-3 expression and degradation of actin. In order to identify the cutting edge future potential of this bio-macromolecule in medicine over the monomer, its in-depth structural and functional properties need to be investigated further.

## Introduction

Clinical and mechanistic research over the past few decades has indicated significant relationships between nutrition and health. The clinical studies with bovine milk derived cancer preventive multifunctional protein lactoferrin (bLf) are currently a promising field of research. Lactoferrin (Lf) is an iron binding ∼78–80 kDa glycoprotein of the transferrin family found to be widely distributed in mammalian milk and most other exocrine secretions such as tears, nasal and bronchial mucous, saliva etc. [1]. Lf comprises of ∼700 amino acids with two symmetrical lobes forming a single polypeptide chain. Each lobe is further sub-divided into two domains that harbor the iron binding sites [2]. In its natural form, native monomeric-bLf (NM-bLf) is approximately 15-20% saturated with Fe^3+^ ions [3]. bLf's role in mammalian iron homeostasis, organ morphogenesis, and bridging innate and adaptive immune functions has resulted in its potential applications in the medical field, along with its wide use as a current nutraceutical and a safe food supplement [1], [4], [5]. More recently, based on the success of animal feeding studies and human clinical trials, bLf has gained significant attention for its prospective use as a safer anti-cancer chemopreventive and therapeutic agent [5], [6], [7].

Because of the worldwide interest in bLf's health and medical applications, investigators for several decades have searched for the most convenient way to produce bLf. Today, native ∼78–80 kDa bLf is mostly produced at a commercial scale from skim milk or whey and bovine colostrum (BC) [4]. When compared to milk, BC is a naturally rich source of bLf, known to contain 1.5–5.0 g L^−1^ of bLf. BC is a thick yellow fluid produced during the first few days after calf's birth. It is known to contain immune, and growth factors to support the growth of the young calf, and also to prevent gastrointestinal infections until the calf develops its own active immune defense [8].

Attempts have also been made to explore the multifunctional nature of Lf. Considering Lf's apparently higher concentrations found in mammalian secretions during the acute phase of infection, inflammation, and its interactions with a range of cells and biomacromolecules (proteins, DNA, oligosaccharides, mononucleotides), a possible role of oligomerization of Lf has been suggested [9]. Earlier, it has been demonstrated that tetramer is the dominating form of human Lf (hLf) found under physiological conditions [10]. Being an acute phase protein with conformational flexibility, Lf can self-assemble into larger structures. However, molecular level explanation for this process is scarce, and investigations are still underway to unravel this property of Lf. Recently, by employing different techniques such as gel filtration, soft laser ablation, small-angle X-ray scattering (SAXS), and light scattering (LS), hLf has been reported to oligomerize into several high molecular weight (HMW) aggregates (70 kDa–800 kDa). The level of oligomerization was reported to depend on the concentrations of Lf, KCl, NaCl and also on the duration of the protein storage in solution [11]. Interestingly, the addition of oligonucleotides, oligosaccharides, or mononucleotides to hLf in the presence or absence of KCl accelerated the oligomerization rate leading to the formation of associates containing ten or more protein molecules. The presence of ions, ATP, NAD, nucleotides, DNA or polysaccharides can further effect the self-association of Lf molecule under physiological conditions [11]. These findings suggest the importance of oligomerization for the multifunctional activities of Lf during host–pathogen interactions, and also in targeting cellular and molecular components of disease signaling pathways.

Chromatographic analysis of bovine milk reveals that the bLf is also found to elute as high molecular weight (HMW) mass complexes corresponding to its monomers, dimers and trimers [12]. Moreover, thermal treatments have been reported to induce bLf aggregation into high molecular weight polymers, and this self-association depends on iron saturation. The thermal stability of bLf markedly increases with iron saturation leading to decrease in the formation of larger aggregates [13]. The oligomerization of bLf therefore can have, varied implications for its biological functions and iron binding abilities. Whether HMW-bLf aggregates/oligomers retain their functional activities like monomer is presently unknown. Due to extensive health promoting activities of monomeric bLf, it becomes essential to unravel the biological functions of HMW-bLf/oligomers of bLf. In this study, we first investigated if it was possible to purify HMW-bLf/oligomers of bLf from BC which is known to contain a high concentration of bLf. We then tested if the HMW-bLf/oligomeric bLf was structurally and immunochemically similar to a native monomeric bLf (NM-bLf). As a proof of concept, by using robust *in vitro* cell bioassays with human breast (MDA-MB-231) and colon cancer (SW480) cell lines from ATCC, we further determined if HMW-bLf/oligomeric bLf potentially targeted cancer cell proliferation and cell death. Another ATCC cell line FHs 74 Int, derived from normal human fetal intestine has been reported to show mature epithelial-like characteristics [14]. It was also employed to investigate the effects of HMW-bLf on normal cells.

## Materials and Methods

### i) Purification of HMW-bLf from bovine colostrum whey

The colostrum sample was obtained from an Australian farm, which was milked normally during postpartum/postnatal period (first 2–4 days after calf's birth). The sample was obtained with the permission from the farm to collect and use it for the study. To obtain the whey proteins, the high viscous colostrum sample was diluted with sterile PBS (pH 7.4). Diluted colostrum sample was skimmed by centrifugation at 3000×g at 4°C for 30 min. The fat (yellow layer) was discarded, and the supernatant was collected and processed immediately or kept frozen at −20°C.

#### Casein removal by acid precipitation

The skimmed diluted bovine colostrum sample as obtained above was acidified by 1 M HCl until it reached pH 4.6 to achieve isoelectric precipitation of casein. The precipitated casein was removed by centrifugation (3500×g for 30 min at 4°C); the supernatant was collected, and the pH was adjusted to pH 7.4.

#### Cation-exchange chromatography

HMW-bLf was further purified using cation exchange chromatography on SP-Sepharose following the modified procedure of Van Berkel *et al.* [15]. Briefly, the column was packed with SP-Sepharose food grade big beads (Amersham biosciences, 17-0657-03). Before first use, the stationary phase was washed with 5 column volumes of water followed by 5 column volumes of 1 M NaCl. It was left in 1 M NaCl for 12 h and then in 5 column volumes of water. The skimmed colostrum after casein removal was diluted in the ratio of 1∶1 with the dilution buffer (0.04 M NaH**_2_**PO**_4_**, 0.8 M NaCl, 0.04% (v/v) Tween 20, pH 7.4), and filtered through 0.45 µm and 0.22 µm filters. The diluted colostrum sample was then loaded in the column and allowed to pass through the column at a flow rate of 0.3 mL min^−1^. Following this, the SP-Sepharose was repeatedly washed with washing buffer (0.02 M NaH**_2_**PO**_4_**, 0.4 M NaCl, 0.02% (v/v) Tween 20, pH 7.4) to remove the unbound whey proteins. Bovine Lf was then eluted with the elution buffer (0.02 M NaH**_2_**PO**_4_**, 1 M NaCl, pH 7.4). The column was run at a flow rate of 3 mL min^−1^. The eluted fractions were dialyzed extensively with a 100 kDa cut off dialysis membrane (Spectrum Labs) against sterile Milli-Q water for 24 h. The purity check and the characterization of eluted fractions were further carried out as described in the following section.

### ii) Characterization of HMW-bLf

#### SDS-PAGE and Western blotting

The purity and molecular weight of purified fractions were analyzed by SDS-PAGE. Following electrophoresis, Western blotting was carried out to confirm the purity and identity of HMW-bLf. The protein was transferred on to a PVDF membrane and probed for bLf with goat anti- bLf antibody (Bethyl Laboratories) diluted in the ratio of 1∶1000 for 1 h at 37°C and secondary anti-goat HRP (Sigma-Aldrich) for 1 h at 37°C with appropriate washing with TBS-T (137 mM NaCl, 20 mM Tris, 0.1% Tween 20, pH 7.6). The membrane was developed using ECL chemiluminescence reagent (GE) in ChemiDoc XRS gel doc (Bio-Rad). Protein samples were freeze-dried for further characterization studies.

#### Dissociation of HMW-bLf into monomers and dimers

In order to identify the homogeneity of HMW-bLf, analysis of the components of HMW-bLf was further carried out by dissociating the complex into its dimeric and monomeric forms in the presence of 1 M NaCl as described earlier for hLf [11]. The study reported that hLf oligomers dissociate fast and almost completely to monomers in the presence of high concentrations (≥1.0 M) of Na^+^ or K^+^. Briefly, 3 mg mL^−1^ of HMW-bLf was dissolved in 50 mM Tris HCl (pH 7.5) containing 1 M NaCl and incubated for 1 day at 37°C. SDS-PAGE was carried out to analyze the dissociation of HMW-bLf complex into its components [11]. Western blotting was carried out to analyze the obtained protein bands by goat anti- bLf (Bethyl Laboratories) diluted in the ratio of 1∶1000 to confirm the presence of lactoferrin.

#### Detection of Lipopolysaccharide (LPS) content

The presence of any LPS activity was measured using E-Toxate assay kit (Sigma-Aldrich). Briefly, 10 mg of purified and freeze-dried HMW-bLf protein was dissolved in endotoxin-free water to prepare a test stock solution of 10 mg mL^−1^, from which serial dilutions of the protein were made to obtain 1, 0.5, 0.1 and 0.01 mg mL^−1^ solutions. Endotoxin standards were prepared from the stock solution (400 EU mL^−1^) to obtain standards of different endotoxin concentrations of 40, 4, 0.4 and 0.04 EU mL^−1^ solutions. Endotoxin free water supplied with the kit was used as a negative control. E-Toxate reagent working solution containing Limulus Amebocyte Lysate (LAL) was prepared according to the manufacturer's instructions. To 0.1 mL of the test/standards/controls, 0.1 mL of E-Toxate reagent working solution was added in 10×75 mm sterile fresh glass tube. The tubes were covered with parafilm and were incubated at 37°C for 1 h without disturbance. After 1 h the tubes were taken out and observed for gelation by tilting them to 120°. The formation of a solid gel was considered a positive result. Semi-solid and watery gels were considered as negative for endotoxin activity. The final endotoxin concentration in the test samples was calculated as; Endotoxin (EU mL-1) = (1/Highest dilution at which the sample was positive)*(Lowest dilution at which endotoxin standard found negative), and it is represented as endotoxin units per mg of the protein.

#### Determination of iron content in HMW-bLf

The iron saturation level in purified HMW-bLf was determined as described earlier by Kanwar *et al.* (2008) [16]. Briefly, to 1 mL of each sample, 50 µL of ascorbic acid was added and allowed to stand for 10 min. Iron standards representing a range of iron concentrations and blank (Milli-Q water) were used to plot a calibration curve. Samples were then centrifuged at 10000 rpm for 20 min. 500 µL supernatant collected from each sample was added into new tubes containing 100 µL of alkaline acetate solution followed by addition of 75 µL of tripyridyl solution. Two hundred microliters of each solution were then transferred into an optically clear 96 well plate, and the absorbance was read at 550 nm. Commercially obtained native LPS free monomeric bLf (NM-bLf) was used to prepare iron saturated (Fe-bLf) and iron free (Apo-bLf) according to the previously described method developed in our laboratory by Kanwar *et al.* (2008) [5], [16]. These forms were used as controls for all the assays.

#### Differential scanning calorimetry (DSC)

5 mg of bLf was measured accurately by sensitive balance and sealed into an aluminum pan. DSC (TA instrument DSC Q200) scans were programmed in the temperature range of 35–110°C and at heating rate of 10°C min^−1^. Native monomeric bovine lactoferrin (NM-bLf) ∼78 kDa was used as a control, along with its iron depleted (Apo-bLf) and iron saturated (Fe-bLf) forms to determine the thermal stability of these proteins.

#### Fourier Transform Infrared Spectroscopy (FTIR) analysis

Samples were mixed with 200 mg of KBr (Sigma- Aldrich) powder and pelleted into a KBr disc using a hydraulic press. FTIR spectroscopy (Bio-Rad with OPUS 5.5 software) analysis was performed between 4000 and 450 cm^−1^ at a resolution of 4 cm^−1^ averaging 10 scans.

#### Gut enzyme intestinal digestion assay

Omnizyme cocktail represents most of the gut enzymes responsible for digestion of proteins, carbohydrates and fats [14]. Omnizyme (Rainrock Nutritionals) enzyme solution was added to the purified HMW-bLf (1∶50) to investigate its stability against gut enzymes. The supernatants collected at different time intervals of (4 h, 6 h and 8 h) were heated at 42°C for 7 min to arrest the enzyme activity and all samples were analyzed by SDS-PAGE.

### iii) Cell bioassays

MDA-MB-231, SW480 and FHs 74 Int cells were obtained from American Type Culture Collection (ATCC, supplied by Cryosite). MDA-MB-231 has been derived from *Homo sapiens* (female) breast carcinoma while SW480 cell line was derived from *Homo sapiens* (male) colorectal adenocarcinoma. Both MDA-MB-231 and SW480 cell lines were epithelial and had adherent growth properties. Both the cell lines were routinely cultured in L-15 media containing 10% FBS at 37°C without CO_2_. FHs 74 Int is a cell line from normal human fetal intestinal tissue, and it was grown in DMEM with 10% FBS at 37°C under 5% CO_2_.

#### Cell cytotoxicity (LDH release) assay

Cytotoxicity caused by treatments with HMW-bLf and other control forms of bLf was measured by release of lactate dehydrogenase (LDH) following cellular injury or cytotoxic insult. The cytotoxicity detection kit (Roche Applied Science) was used according to manufacturer's instructions. The assay is based on calculating the LDH leakage into the culture medium after 24 h following exposure of cells to different treatments. LDH is constitutively present in all cells and is released into supernatant due to cell membrane damage. MDA- MB-231 SW480 and FHs 74 Int cells were treated with media containing different treatment concentrations (800, 1600, 2400, 3200 µg mL^−1^) of HMW-bLf for 24 h. Each treatment was carried out three times, in triplicates. The absorbance values were measured by using a SH-1000 lab absorbance microplate reader (Corona Electric) at 492 nm with reference wavelength at 620 nm. All values were the product of background subtraction with media alone reacting to the LDH reagent. Addition of Apo-bLf, Fe-bLf and HMW-bLf to the media did not alter the background reading. The % cytotoxicity was calculated by the formula; Cytotoxicity % = [(Exp. value-Low control)/(High control-Low control)] ×100.

Negative control (Low control) used was the cell culture supernatant of untreated cells and positive control (High control) representing the maximum value of LDH release was the cell culture supernatant of the cells treated with 1% (v/v) Triton X-100 (Sigma-Aldrich, Sydney, Australia). All obtained values for treatments are represented relative to untreated control value set to zero.

#### Cell proliferation assay

The inhibition of cell proliferation caused by bLf treatments was analyzed by determining the DNA content of the cell using CyQuant assay kit (Invitrogen) as per manufacturer's instructions. Briefly, viable MDA-MB-231 and SW480 cells were plated, initially at a concentration of 2×10^5^ cells/mL in 96 well microplates and incubated overnight. Cells were then treated with fresh media containing different treatment concentrations (800, 1600, 2400, 3200 µg mL^−1^) of HMW-bLf for 24 h and the media was aspirated out. The CyQuant reagent was then added to the cell pellet, and the corresponding fluorescence from the DNA was measured using a fluorescence reader at an excitation wavelength of 490 nm and emission of 530 nm. Treatments with different doses of HMW-bLf were carried out in triplicates, and the assay was repeated three times. Media with 20% FBS was used as a positive control. Background measurements for the plate alone with CyQuant reagent were subtracted from the test values.

#### Measurement of cell death by Flow cytometry

MDA-MB-231, SW480 and FHs 74 Int cells were treated with different concentrations of HMW-bLf for 24 h, and then trypsinized. Cell pellets were washed with sterile PBS and resuspended in 500 µL sterile PBS containing 0.1 mg mL^−1^ Propidium Iodide (PI) solution. PI is a fluorochrome that intercalates into double-stranded nucleic acids. After 15 min of incubation in dark at room temperature, the cells were analyzed for cell death (viability counts) using Flow cytometry (BD FACSCanto II). Untreated unstained cells were used to set up the instrument for acquisition, and the gating was adjusted to have <1% PI positive cells. The test samples were acquired after this under the same settings, and the percentage of cells in the PI positive gate was considered as the percentage dead cells.

#### Cellular internalization of HMW-bLf

Cellular uptake of HMW-bLf was studied by immunofluorescence and visualized by confocal microscopy. SW480, MDA-MB-231 and FHs 74 Int cells were seeded in an 8-chamber multi-well slide (BD falcon) at a density of 1×10^5^ cells/well and were then treated with 800 µg mL^−1^ of HMW-bLf for different time intervals in assay media (basal cell media with 1% FBS). Cells (to be treated as well as the untreated) were pre-conditioned to the assay media for 24 h before the actual assay by culturing in the assay media and remained healthy. Following treatment, the medium was removed, and the cells were washed thoroughly using PBS (pH 7.4) to remove unbound and non-internalized HMW-bLf from the cell layer, followed by fixation with 4% paraformaldehyde. After fixation, the cells were permeabilized with 0.1% TritonX-100 for 5 min on ice. Blocking was carried out with 2% sterile rabbit serum in PBS and cells were then incubated with primary antibody, goat anti-bovine lactoferrin (Bethyl Laboratories) at a dilution of 1∶200 in PBS at 37°C for 1 h. The primary antibody was then removed and after washing, cells were incubated with anti-goat IgG-FITC conjugate (Sigma-Aldrich) and counterstained for actin with Phalloidin-AlexaFluor 568 (Invitrogen) and nucleus with DAPI in fluorshield (Sigma-Aldrich). The slides were imaged using TCS SP5 Leica broadband confocal microscope and processed using LAS-AF software. Media containing 1% FBS was used to avoid any protein-protein interactions during treatments. Because we used the lower FBS concentration in the assay media than the normal growth media, the cell viability was checked with trypan blue exclusion assay, before performing cellular uptake studies. No difference was noted between the cells ability to exclude the trypan blue dye when cells were grown in media containing 10% FBS or of that in 1% FBS for 24 h (Figure S1 in File S1). The 800 µg mL^-1^ of HMW-bLf concentration was used for its comparatively lower cytotoxic effects on cancer cells. As a result following incubation with these treatments, lesser cell detachment (of dying/dead cells) was observed from the slide, and the cell monolayer survived the staining and imaging procedure.

#### Caspase-3 assays

Activation of cellular apoptosis was determined by caspase-3 activation assay using the method described by Fujie *et al.* [17]. Cells were treated with different concentrations of HMW-bLf and Fe-bLf (3200 µg mL^−1^) for 24 h. Cells were lysed, centrifuged, and supernatant containing 100 µg mL^−1^ protein lysates were taken for analysis from each treatment. 50 µL of mixture reagent (Dithiothreitol (DTT) in radio-immunoprecipitation assay RIPA buffer solution) was added. Finally, 6 µL of substrate acetyl-Asp-Glu-Val- Asp p-nitroanilide (Sigma-Aldrich) dissolved in dimethyl sulfoxide (DMSO) at 10 mg mL^−1^ was added to all samples. The plate was then incubated for 180 min at room temperature in dark. Level of caspase-3 expression was quantified by using SH-1000 lab absorbance microplate reader (Corona Electric) at 405 nm.

The activation of cleaved caspase-3 was also confirmed using Western blot for cleaved caspase-3. Cells were treated with different concentrations of HMW-bLf, Apo-bLf and Fe-bLf (3200 µg mL^−1^) for 24 h. Cells were lysed using RIPA (Radio Immuno-Precipitation Assay) buffer, and 75 µg of total protein was loaded for the SDS-PAGE. The proteins were then transferred to a PVDF membrane using Trans-Blot Turbo (Bio-Rad) semidry transfer instrument. The membrane was blocked using 3% skim milk for one hour after which they were probed using cleaved caspase-3(Asp175) primary antibody (Cell Signaling Technology) in the dilution of 1∶500 at 37°C for 1 h. The primary antibody was then removed, and the membranes were washed thrice with TBS-T to remove unbound primary antibodies. It was then incubated with 1∶40000 anti-rabbit HRP secondary antibody (Sigma) for 1 h at 37°C. After incubation, the secondary antibody was removed and membrane was washed three times with TBS-T. The membrane was developed using ECL chemiluminescence reagent (Amersham) and viewed under Chemi-doc XRS gel documentation system (Bio-Rad).

#### Statistical analysis

Data was expressed as mean values (±SD) and Student's t-test was performed for evaluating statistical significance. A value of (p<0.05) denotes statistical significance, whereas (p≤0.01) denotes results that are highly significant. All treatments were tested in triplicate, and each assay was repeated 3 times.

## Results and Discussion

**Figure 1:**
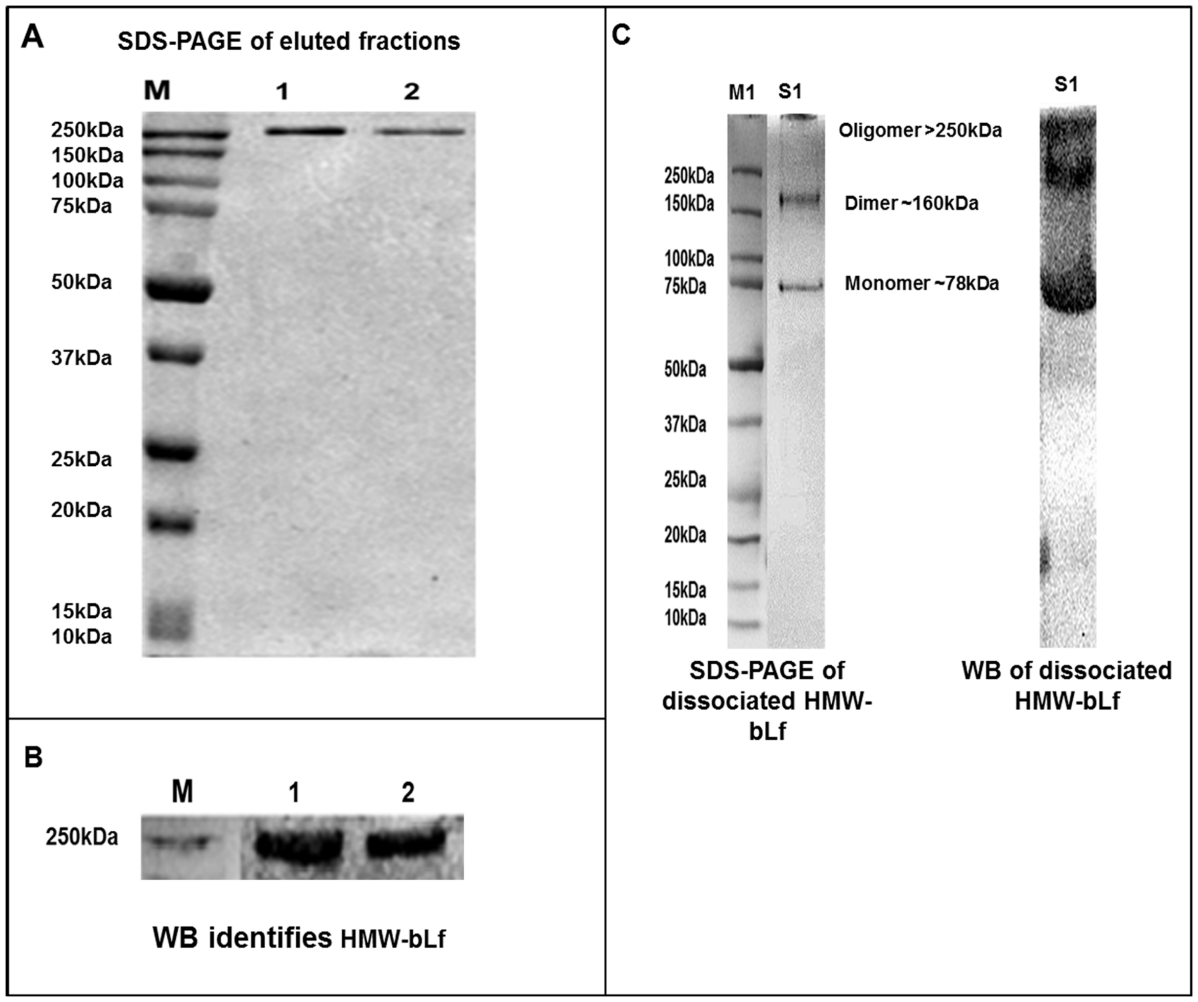
Purification and analysis of components. A) SDS-PAGE analysis of purified HMW-bLf indicating the presence of pure ≥250 kDa protein in elutes (lanes 1 and 2). B) The purified protein was confirmed to be bLf through Western blotting using anti-bLf specific antibody. C) Dissociation of HMW-bLf in 1 M NaCl into the dimeric (∼160 kDa) and monomeric (∼78 kDa) forms (lane S1). These were confirmed to be bLf bands by Western blot for the same dissociated sample (S1). The absence of any other lower bands and the detection of all the constituent bands by anti-bLf specific antibody indicate that HMW-bLf is an oligomer formed by the interactions of monomeric bLf molecules.

HMW biomacromolecular complex containing bLf was purified from skimmed defatted, casein free colostrum whey using cation exchange chromatography with SP Sepharose food grade big beads. Casein from skimmed bovine colostrum was completely precipitated at lower pH and was removed. The whey obtained was therefore casein and fat free. Lactoferrin has an Iso-electric point of pH 8.7 which is the highest among all the milk proteins. Hence bLf remains positively charged even at the near neutral pH of 7.4. It binds very strongly to the cation exchange resin whereas other whey proteins are not strongly bound, and they get washed off during the washing step, leaving only the bLf molecules attached to the resin. This bound bLf was then isolated using a strong cationic 1 M salt solution [18]. The eluted protein fractions were then subjected to extensive dialysis. The purity of the eluted fractions was checked by SDS-PAGE that showed a single HMW band ≥250 kDa (Figure 1A). The HMW protein band on SDS-PAGE was later identified as bLf by Western blotting using anti-bLf antibody (Figure 1B). Native bLf as a monomer has a molecular mass of ∼77–80 kDa, depending upon the glycosylation of mature protein, the ≥250 kDa HMW-bLf therefore indicates a trimer or a probably partially degraded tetramer due to its possible interaction with Sepharose resin during purification. This phenomenon has been reported earlier that incubation of hLf with oligosaccharides led to the formation of unstable oligomers as studied by gel filtration chromatography using polysaccharide resins such as Sephadex, Sepharose 4B, etc. [11]. These authors suggested that Sepharose, containing alternating residues of β-D-galactopyranose and 3, 6 anhydrido-α-1-galactopyranose linked by 1-4 bonds, could dissociate hLf oligomers. As explained above, Lf has high affinity for such resins, thereby efficiently interacts with them. Lf bound to these gel filtration/cation exchange chromatographic resins can be eluted at high salt concentrations (KCl or NaCl), since the high ionic strength causes dissociation of oligomers, and Lf then can be mostly eluted as a monomer.

Interestingly, previous Sephadex G-200 chromatographic purification reports reveal that when the concentration of bLf was high in mammary secretions due to infections such as during mastitis, bLf was initially obtained as 77 kDa monomer, and as the infection progressed, it was separated at approximately the size of a trimer (∼240 kDa). These observations were therefore concurrent with the increasing bLf concentrations during infections [19]. Another study similarly reported that the bLf, in non-lactating mammary secretions during involution, was mainly present as HMW complexes rather than as monomers, and majority of total bLf existed in ∼250 kDa molecular mass fractions [12]. Structural studies employing the method of sedimentation equilibrium in the ultracentrifuge, on purified preparations of bLf obtained from commercial preparations, revealed that the purified protein exhibited heterogeneity with respect to its molecular weight in dilute aqueous salt solutions. The top of the cell had 76 kDa bLf material while >200 kDa material was sedimented at the cell bottom. These findings implied that bLf associated in the native state in a concentration dependent manner, and aggregates as high as trimers were obtained [20]. The nature of these intermolecular interactions in bLf oligomers remained unknown, and mostly investigators in the late last century, did not take bLf oligomeric forms into consideration. There are reports that bLf dimers have also been mistaken for IgG, because of its molecular weight that is twice of the bLf monomer [21], [22].

More recently, emerging experimental evidence indicate Lf as an extremely conformationally dynamic protein that is prone to self-association. The macromolecule has a dumbbell shape, well described by a bi-axial ellipsoid with half-axis of 47 Å and 26 Å [11], [13], [21]. While the levels of self-association were shown to depend on the number of conditions such as Lf concentration, presence of salts, ligands, storage in solutions, iron saturation and temperature, a molecular level explanation remains yet to be understood for this phenomenon. Without salt or at physiological salt concentrations, bLf as well as hLf reportedly self-associate in aqueous solutions as dimers, trimers, and also as tetramers with tetramer being the dominant form [11], [21]. In these studies, gel filtration analysis also revealed small peaks of the decamer. For tetramer formation, which is the predominant molecular form of Lf in human serum, tears, and breast milk, calcium dependent oligomerization of hLf has been reported, [23]. Therefore the purification of ≥250 kDa bLf oligomer in our study is in agreement with the aforementioned findings of other investigators. Colostrum is known to contain higher concentrations of bLf and calcium ions than milk [8], [24], [25] these could have also contributed to the self -association of bLf into HMW oligomeric complex along with the unidentified molecular trigger(s) of self-association.

HMW- bLf molecule was also found to be dissociated into the dimeric (∼160 kDa) and monomeric (∼78 kDa) forms of bLf when kept for 24 h in the presence of 1 M NaCl. This observation is inconsistent with earlier findings on hLf [11]. As determined by gradient SDS-PAGE (reducing gel) analysis followed by Western blotting with anti-bLf antibody (Figure 1C), all the three bands were identified as bLf protein. More interestingly, a progressive increase in the intensity of monomeric ∼78 kDa band with a simultaneous decrease in the remaining HMW-bLf band was observed. This is clearly evident in Western blot (Figure 1C). We have also noted that since the purified HMW-bLf sample (Figure 1B) was stored for about a year after purification and lyophilization, a much larger bLf aggregate (polymer) in the stacking gel was identified that showed lower mobility protein material with a comparative decrease in its band density, than the ≥250 kDa oligomeric complex band observed just after the purification (Figure 1A). These observations are also consistent with the earlier oligomerization studies on hLf which report that in the presence of 1 M NaCl, the hLf oligomers dissociate slowly into monomers whereas, during storage of dialyzed and lyophilized protein solutions at neutral pH, the monomeric or oligomeric forms slowly aggregate[11], [26]. We also performed Western blotting for bovine IgG to identify dimeric −160 kDa band, and the larger HMW-bLf aggregate band but no immunoreactivity was observed indicating there was no contamination from IgG. Further, no other bands that could correspond to molecular weights of any other milk protein (lysozyme − 14.6 kDa, α-lactalbumin -14.12 kDa, β-lactoglobulin – 22.40 kDa, αs_1_-casein – 33.30 kDa, β-casein – 37.50 kDa) in the dissociated sample of HMW-bLf were observed on SDS-PAGE reducing gel (Figure 1 C), indicating their absence in the sample.

The absence of any LPS contamination and endotoxin activity in the purified protein was confirmed using E-Toxate assay kit which indicated the presence less than 0.04 EU/mg (Endotoxin Units/mg) of endotoxin activity in the purified HMW-bLf samples. This was much lower than the FDA accepted standards [27]. Our observations thus indicate that purified HMW-bLf could be made up of bLf molecules forming a multimeric complex due to non- covalent, ionic interactions. These interactions were broken in the presence of strong ionic solution (1 M NaCl) and results in the appearance of more intense low molecular weight monomeric as well as dimeric forms. In the existing literature, despite the emerging data on Lf oligomerization/self-association, little is known about the nature of the chemical linkages/binding interactions that result in the formation of Lf-Lf complexes. Ionic, thiol/disulfide and hydrophobic interactions may all be involved in these intermolecular interactions. Earlier findings on thermal aggregation of bLf molecules proposed that the bLf aggregation proceeded via a combination of non-covalent interactions and intermolecular thiol/disulphide reactions that did not require free thiol residues. Specifically, the thermal aggregation of iron saturated bLf was mainly driven by non-covalent interactions, with intermolecular thiol/disulphide reactions also observed above 80°C [13]. Using force field based molecular modeling of the protein–protein interaction free energy, it was demonstrated that at neutral pH, Lf forms highly stereo-specific dimers. This self-association is driven by a high charge complementarity across the proteins' contact surface [21].

**Figure 2:**
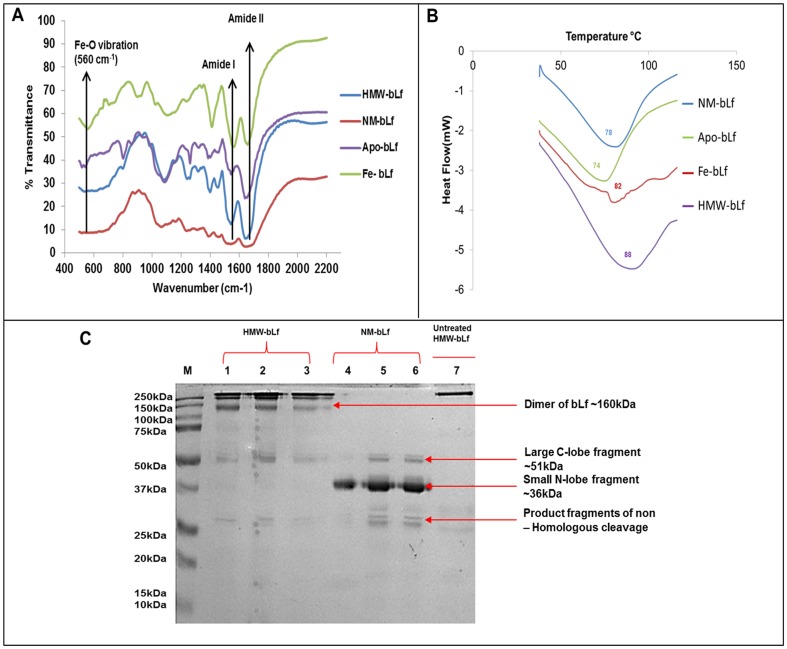
Physico-chemical characterization of HMW-bLf. A) Fourier transform infra-red (FTIR) spectra of HMW-bLf indicating characteristic peaks and compared with other forms of bLf. B) Differential scanning calorimetry thermograms of the different bLf forms C) SDS-PAGE showing the comparative resistance of HMW-bLf to Omnizyme (human digestive enzyme cocktail) treatment. It also gives an indication that HMW-bLf is an oligomer made of bLf monomers; because upon digestion HMW-bLf besides dimers and trimers does not release any other fragments lower than ∼78 kDa apart from the ones produced from the digestion of commercially pure NM-bLf.

Because of its iron binding properties, Lf is known to exist in two forms: holo-Lf (binds two Fe**^3+^** ions, iron saturated), Apo-Lf (iron depleted) [2]. Native Lf is only partially saturated. Therefore, we measured the iron content of HMW-bLf and found that it contained only 0.47% iron and thus, was more like Apo-bLf (1.1%), when compared with other forms of bLf such as NM-bLf (22% iron), and iron saturated bLf (Fe-bLf) >94% iron (Figure S2 in File S1). Figure 2A shows a comparison between the FTIR spectra of HMW-bLf, NM-bLf, Apo-bLf and Fe-bLf. The characteristic amide carbonyl stretching appeared between 1630 and 1650 cm^−1^, the C-N stretch was observed between 1500 and 1550 cm^−1^ while the O-C-N bend was discernible between 675 and 721 cm^−1^ in the four forms. The Fe-O vibration band appeared at 560 cm^−1^ in the FTIR spectrum of Fe-bLf while it was not pronounced in the other three spectra suggesting high iron content in Fe-bLf (Figure S3 in File S1). This also confirms our iron content estimation results. bLf is classified as a glycoprotein, the bands from 900–1200 cm**^−1^** due to C–O, C–C, C–O–H, C–O–C vibrations of the carbohydrate moiety were therefore observed in all the four forms of bLf [28].

Exploring the thermal stability of bLf has been also important because of its bioactivity. In order to develop a practical method for pasteurization of bLf, the heat stability has been studied previously. Several factors can affect the heat stability of bLf such as pH, salts, and other whey proteins [29]. We tested the thermal stability of HMW-bLf, Fe-bLf, Apo-bLf and NM-bLf in lyophilized powder form by DSC. The thermogram of ≥250 kDa HMW -bLf (Figure 2B) showed denaturation peak at 88°C, which was the highest when compared with those of Fe-bLf, Apo-bLf and NM-bLf, and their denaturation peaks were observed at 82°C, 74°C and 78°C, respectively. These findings suggest that the higher thermal stability of HMW-bLf may be due to its structural integrity as an oligomer. Among other bLf forms, the Fe-bLf was comparatively more resistant to heat when compared to the Apo-bLf and the NM-bLf. Similar findings have also been reported, suggesting that an increase in protein stability depends upon the degree of iron saturation [13], [30], [31]. Increased thermal stability of Fe-bLf (holo-bLf) has been attributed to the more compact conformation, adopted by the molecule by binding a ferric ion in the inter-domain cleft of each lobe [2]. Slightly higher thermal stability of the NM-bLf than the Apo-bLf could thus be attributed to partial iron saturation status of the native protein.

From our results, and the aforementioned discussion, it can be inferred that the interactions between the bLf molecules may largely be ionic to form the HMW-bLf oligomer. Oligomerization/protein aggregation is a concentration dependent process. At high concentration of Lf and of calcium in the bovine colostrum, the equilibrium shifts towards the formation of oligomers, however, under dilution or at high Na^+^/K^+^ concentration the ionic bonds are broken and they tend to shift towards existence as monomers. We propose that HMW-bLf, which is similar to Apo-bLf and lacks mostly the iron content, and likely contain lot of free aspartate and tyrosine residues. This confers a very open, flexible configuration to HMW-bLf molecule similar to Apo-bLf [32]. Due to this, the ability of the molecule to undergo intermolecular interactions was highly increased when maintained at a high concentration and in the absence of any salt content, especially iron [13]. These complex interactions in HMW-bLf appeared to be very strong [11] and led to the formation of even larger aggregates upon prolonged storage (Figure 1C). We have also noticed that NM-bLf (22% iron saturated), when stored at high concentration, displays trimers and dimers formation visualized on denaturing SDS-PAGE condition (data not shown). In a study that investigated the effect of iron saturation on thermal aggregation of bLf in Apo state, it was shown that Apo-bLf associated into large polymers by non-covalent interactions without the participation of disulphide cross-links. The more unfolded structure of heat sensitive Apo-bLf may have increased the exposure of non-covalent sites normally buried in the core of both lobes of the protein, thereby favoring intermolecular interactions and the formation of larger aggregates [13]. The in-depth molecular and biophysical characterization of the HMW-bLf needs to be investigated further with more powerful proteomics tools such as Mass spectroscopy and Circular Dichroism, in order to identify the chemical as well structural changes/linkages in the oligomer. To determine spontaneous association of bLf in physiologically simulated solution SAXS and LS can be employed to analyze the oligomeric states of HMW-bLf.

The consumption of test drinks containing Apo-bLf and iron saturated bLf in human volunteers has shown that Apo-bLf is more susceptible to *in vivo* gut digestion, than the corresponding iron-saturated form [33]. Similarly, we have reported earlier the resistant nature of Fe-bLf towards Omnizyme (a digestive enzyme cocktail) [5]. In the current study, HMW-bLf was found to be more resistant to Omnizyme digestion *in vitro*, even after 4, 6, and 8 h incubation periods. Figure 2C shows that HMW-bLf in lanes 2, 3 and 4 showed excellent stability to digestion, with digested forms appearing as 150–160 kDa dimers at various intervals of time (4, 6 and 8 h) while faint bands at ∼78, 50 and 25 kDa. In the case of NM-bLf, in lanes 5, 6 and 7 the 78 kDa bands have completely disappeared and digested to its peptides that appeared at 51, 37and ∼25 kDa. HMW-bLf's comparatively much stronger resistance to gut enzyme digestion despite having far lower iron content than NM-bLf (0.47% versus 22%).This can be explained in terms of the robustness of its structure being a larger biomacromolecule/oligomer. This property will therefore prove beneficial for its potential use as a nutraceutical. In that case, if given orally there will be an increase in the *in vivo* bioavailability of HMW-bLf to the required sites of the action in the body, e.g., tumor/infected and inflammatory tissues as compared to NM-bLf, which is comparatively more prone to digestion. This assay also gives an insight into the components of HMW-bLf and indicates that HMW-bLf appears not to be made up of any other component than that of NM-bLf. The fragments that are generated by the Omnizyme digestion show that NM-bLf (Lanes 4, 5 and 6) can be degraded into a large C- terminal lobe with a part of the N-terminal region and the connector seen as a 51 kDa band. The C-terminal lobe on further digestion produced the band at 36 kDa. This is not a homologous digestion, and it does not produce two equal sized fragments hence resulting in a few smaller fragments seen at 25 kDa [34]. The observation that below ∼78 kDa, the HMW-bLf also produces the same set of bands as that of NM-bLf (Figure 2C, Lanes 1–3 and 4–6 respectively) thus is in agreement with our dissociation findings of HMW-bLf (Figure 1C) that it was comprised of bLf monomers.

We have shown earlier that 100% iron-saturated bLf form (Fe-bLf) when given orally to mice prior to chemotherapy caused its augmentation [16]. Significant eradication of large tumors in combination with anticancer drugs was observed. However, 20% iron-saturated (NM-bLf) or Apo-bLf remained ineffective in eradicating these tumors, owing to their high degradation in the gut as compared to Fe-bLf. In order to increase the more bioavailability of Fe-bLf to the tumour sites, more recently, we have developed a novel nanodrug delivery system (alginate-enclosed chitosan–calcium phosphate-loaded Fe-bLf nanocarriers) for oral delivery. By employing human colon xenograft model, we reported that nanoformulated Fe-bLf when fed orally led to the complete inhibition of tumorigenesis in prevention mode. A complete tumor rejection through regression, in the treatment mode was also observed [5]. These nanocarriers thus led to the increased bioavailability of Fe-bLf to the tumor sites, and were found to be safe and nontoxic. Similarly, another study has shown that the anti-tumor effects of NM-bLf on melanoma cells can be enhanced with liposomalization. A lipid delivery system (liposomes) was used to prevent the NM-bLf from proteolysis or neutralization by serum proteins [35]. Considering the findings that tetramer is reported to be the dominating form of hLf observed under physiological conditions [10], [23], and bLf self-associates as dimers, trimers and tetramers in mammary secretions during infection and involution [12], [19], it may imply that the physiological existence of Lf oligomers, can therefore be a protection strategy acquired by multifunctional Lf against proteolysis or neutralization by serum proteins.

**Figure 3:**
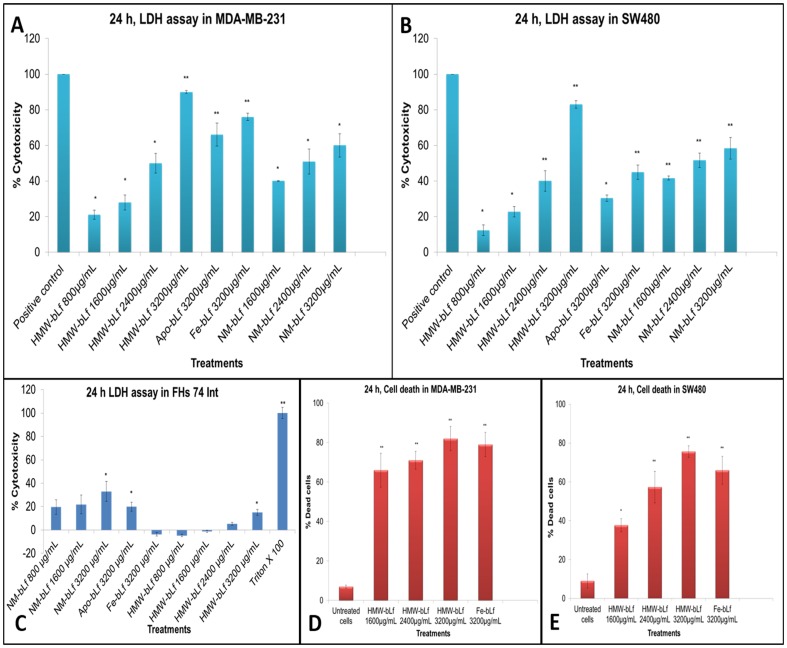
Cytotoxic effects of HMW-bLf. A B and C represent the cellular cytotoxicity measured by LDH release assay induced by HMW-bLf in a concentration dependent manner, in MDA-MB-231 (human breast carcinoma) SW480 (human colorectal adenocarcinoma) and FHs 74 Int (normal intestinal cells) cells. D and E show the cell death (mortality count) as measured by Flow cytometry using propidium iodide staining (* *p<0.05* and ** *p<0.01*). Other forms of bLf were used for comparison.

Earlier it has been shown that NM-bLf decreased the viability of breast cancer cell lines HS578T and T47D by inducing a 2-fold increase in apoptosis, and decreased the proliferation rates as well in both the cell lines [36]. A similar effect of bLf was seen on colon carcinoma [37] and *in vivo* on tumors of melanoma, EL-4 T-cell thymic lymphoma and Lewis lung cancer cells [16]. To test the anticancer activities of HMW-bLf, we employed MDA-MB-231 (human breast carcinoma) and SW480 (human colorectal adenocarcinoma) cell lines. Figure 3 (A and B) shows the cytotoxic effects of HMW-bLf. It was assessed by measuring the leaked LDH enzyme (as a cell viability biomarker), from dying or dead cells (early/late apoptotic and necrotic) due to their damaged cell membranes. HMW-bLf was found to be effective in a concentration dependent manner in inducing cell cytotoxicity in both MDA- MB- 231 and SW480 cells. The cytotoxicity values of HMW-bLf and Fe-bLf at the highest concentration used (3200 µg mL^−1^), were highly significant (*p<0.01*) with 90% and 76% cytotoxicity observed respectively. Similarly, when compared with the other control forms of bLf, 3200 µg mL^−1^ of HMW-bLf also showed significantly highest cytotoxicity in SW-480 cells. Though, among all the concentrations of HWW-bLf tested on SW480 cells, it showed lowest cytotoxicity values at a concentration of 800 µg mL^-1^ but the effect was still statistically significant (p<0.05) when compared to the untreated cells' (value set at zero). The cytotoxicity of HMW-bLf towards non-cancerous human cells (of normal intestinal origin) was also tested by treating FHs 74 Int cells with its different concentrations. A significant increase in the LDH release activity corresponding to 15% cytotoxicity was observed only at the highest concentration (3200 µg mL^−1^) of HMW-bLf, as shown in Figure 3 C. More importantly, in HMW-bLf treated colon cancer cells (SW480), the corresponding cytotoxicity values at all the concentrations tested were significantly higher (P<0.01) than those obtained with FHs 74 Int cells. Moreover, at HMW-bLf treatments(800 µg mL^−1^, 1600 µg mL^−1^ and 2400 µg mL^−1^), LDH release activity from FHs74 Int cells was significantly lower than that of untreated cells. The presence of Lf in mammalian milks and bovine colostrum has an important role for the normal gut cell growth, maturation and repair in young ones. It could thus possibly be a therapeutic action of the HMW-bLf in repairing membrane damage, occurred normally to these normal intestinal cells under *in vitro* culture. A maximum of LDH release corresponding to 33% cytotoxicity (p<0.01) was seen with NM-bLf 3200 µg mL^−1^ treatment to FHs74Int cells. There was no significant difference observed among the cytotoxic activities of Apo-bLf (20±6.5%), NM-bLf (33±15%) and HMW-bLf (15±4.2%) at 3200 µg mL^−1^. However, when compared at 1600 µg mL^−1^, NM-bLf caused significantly higher cytotoxic effect on FHs74 Int cells than HMW-bLf at the same concentration. It is important to note here that among all the treatments, NM-bLf induced highest 33% cytotoxicity to the normal intestinal cells. The non-toxicity of bLf to normal cells/tissues during long-term feeding has been clearly shown through number of *in vivo* animal studies, and oral feeding trials in human volunteers and colon cancer patients [4], [5], [6], [16]. It has been approved by the Food and Drug Administration (FDA) of United States in 2001, and later by European Food Safety Authority as a dietary supplement in food products [38], [39]. Moreover, FHs 74 Int is a fetal derived intestinal cell line, known to grow as normal enterocytes. Considering Lf's intense affinity for iron [1], [32], it can be explained that FHs 74 Int cells' requirement of iron, for their cell viability, appears to be targeted by both Apo-bLf (iron free) and NM-bLf (partially saturated with iron) thus showing significantly higher cytotoxicity values than Fe-bLf. On the other hand, HMW-bLf although showed very low iron content but having a more robust complex molecular structure, might not bind iron as fiercely as Apo-bLf and NM-bLf do. Therefore, it showed comparatively less cytotoxicity than Apo-bLf and NM-bLf. These observations need follow-up investigations to determine the complete safety profile of HMW-bLf towards the normal cells.

Since recent reports suggest that LDH release measurements can underestimate the cytotoxicity caused by compounds causing cell cycle arrest [40], the cytotoxic effects of HMW-bLF were also studied by Flow cytometry using PI staining. Figures 3 (D and E) and Figure S4 in File S1, show the cell death induced by HMW-bLf in cancer cells and FHs74 Int cells, respectively. PI test values appeared higher (Figure 3 D and E) than the LDH release assay (Figure 3 A and B). However, the results of the two assays were in general agreement and showed that HMW-bLf targeted cell death in a dose-dependent and cell specific manner. Even within cancer cells from different tissues, it was significantly more cytotoxic towards MDA-MB-231 cells than SW480 cells.

**Figure 4:**
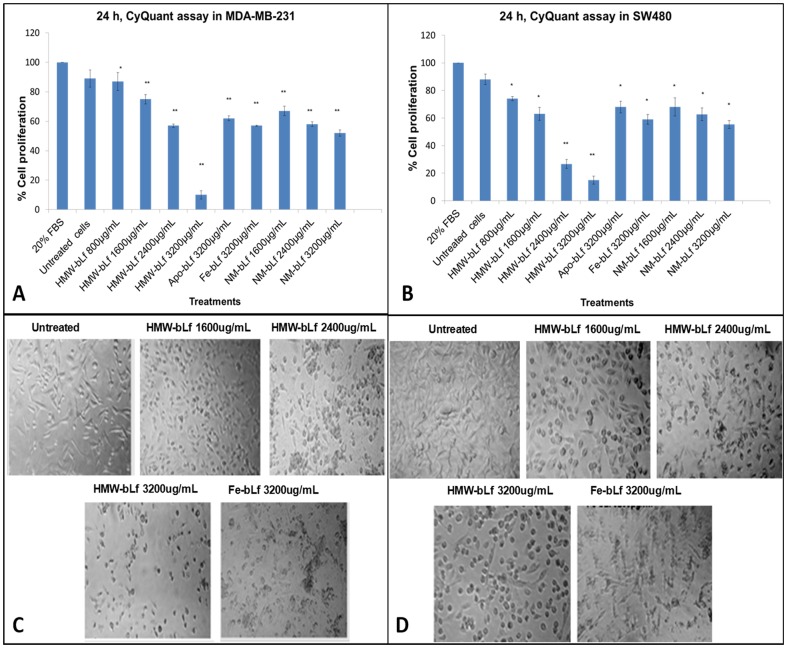
Cell growth inhibition. HMW-bLf decreases the cellular proliferation of MDA-MB-231 and SW480 (A and B respectively) cells in a concentration dependent manner. The cell fate was also monitored by analyzing the cell morphology (C – MDA-MB-231 and D – SW480) which clearly indicates cell death. (* *p<0.05* and ** *p<0.01*)

To determine the growth inhibitory properties of HMW-bLf, we further assessed the proliferation of MDA-MB-231 and SW480 cells after treatments for 24 h using the CyQuant assay. Figure 4 (A and B) shows that HMW-bLf decreases the rate of cell proliferation in a dose-dependent manner. At the highest dose, it significantly (*p<0.01*) inhibited the proliferation of both MDA- MB- 231 and SW480 cells (up to 90%), a better rate than any other control forms of bLf. Furthermore, observation into the effects of HMW-bLf on cell morphology revealed that cells after treatments for 24 h, exhibited poor growth showed altered morphology with high levels of cellular fragmentation and apoptotic bodies. Both cell lines used in this study showed detachment from culture dish bottom and floating dead cells were mostly observed, when highest concentration of HMW-bLf was employed (Figure 4 C and D).

**Figure 5:**
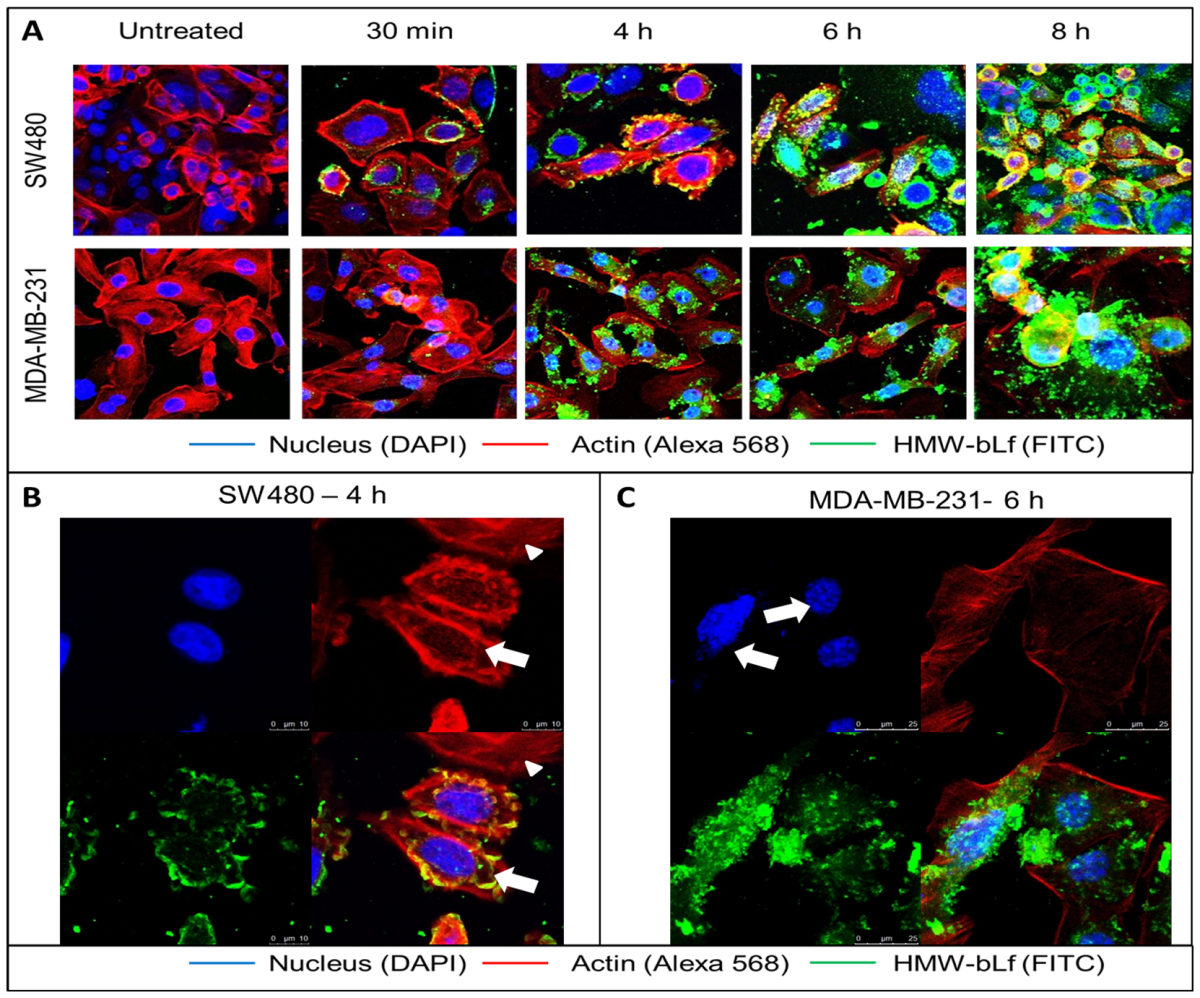
Cellular uptake of HMW-bLf. A – Representative confocal microscopic images show HMW-bLf internalization in MDA-MB-231 and SW480 in a time dependent manner. The degradation of actin an indicator of apoptosis was observed after 6 h of treatment with HMW-bLf. The reduction in the intensity of the Alexa 568 signal indicates the degradation of the actin cytoskeleton. B and C are high magnification images HMW-bLf internalization in SW480 (B) and MDA-MB231(C) with separate panels showing nucleus, actin and HMW-bLf alone. Arrows in 4B points out to the cells that have taken up HMW-bLf showing perturbed actin structure, and arrowhead points out to the cell with intact actin structure and is without HMW-bLf uptake in 4 h (SW480). Arrows in 4C point out to the beginning of nuclear degradation at 6 h (MDA-MB-231).

NM- bLf is known to be internalized into live cells through cell surface (membrane) receptor mediated endocytosis mechanism [1], [41]. The published data report that transferrin receptor (TfR), lactoferrin receptors and low density lipoprotein receptor-related protein receptors (LRPs) play a crucial role in facilitating the internalization of bLf inside cells[42], [43]. Using both colon and breast cancer cells it was observedthat bLf internalized into the membrane, cytoplasm and nucleus in a time dependent fashion (unpublished studies from our laboratory). Therefore immunofluorescence was carried out in order to determine whether the oligomerization state of HMW-bLf affects its cellular internalization and receptor binding properties using MDA-MB-231 SW480 and FHs74 Int cells (Figure 5 and Figure S5 in File S1). Since the cytotoxicity of HMW-bLf was observed in a concentration dependent manner, only lowest concentration (800 µg mL^−1^) was used. Therefore following incubation, lesser cellular detachment (of dying/dead cells) was observed from the slide making the immunostaining and imaging possible. The confocal microscopy images indicate that beginning at 30 min after incubation, there was a rapid internalization of HMW-bLf by the three cell types. The presence of green fluorescence signal of bLf specific antibody immunostaining was mainly seen on the cell surface and cell membrane. Since internalization is a time dependent process, it was more evident at earlier time points of incubation (30 min and 4 h). HMW-bLf was also found to be localized along the perinuclear region in 4 h. In the images obtained after 6 h and 8 h of incubation periods, HMW-bLf was seen to be internalized into the nuclei of cancer cells. This indicates that bLf in its oligomeric state retains its ability to interact with receptors and is taken up by the cells in time dependent fashion, although further investigations are needed to determine the receptor-ligand interactions completely. Taken together, the results of cellular internalization, LDH release and CyQuant assays reveal that cellular uptake of HMW-bLf even at 800 µg mL^-1^ proved effective; where internalized HMW-bLf displayed its functional bioactivity in terms of inducing significant cytotoxicity (LDH release) and anti-cell proliferative activity in cancer cells. Interestingly, the degradation of actin network within the cells was observed by the reduced fluorescence intensity of phalloidin - AlexaFluor 568 stain (red fluorescence). This shows that the cellular uptake of HMW-bLf triggers the process of apoptosis resulting in the loss of actin framework which acts as a substrate for caspase-3 and its downstream products [44]. Figure 5B shows the high magnification image of HMW-bLf internalization in SW480 cells after 4 h with the arrows pointing out at the cells that have internalized HMW-bLf and thereby showing the degradation of actin. An arrow head points to the cell with intact actin structure that has not taken up HMW-bLf. Figure 5C is a high magnification image of HMW-bLf internalization at 6 h showing the beginning of nuclear material degradation which is the final stage in the apoptotic pathway.

**Figure 6:**
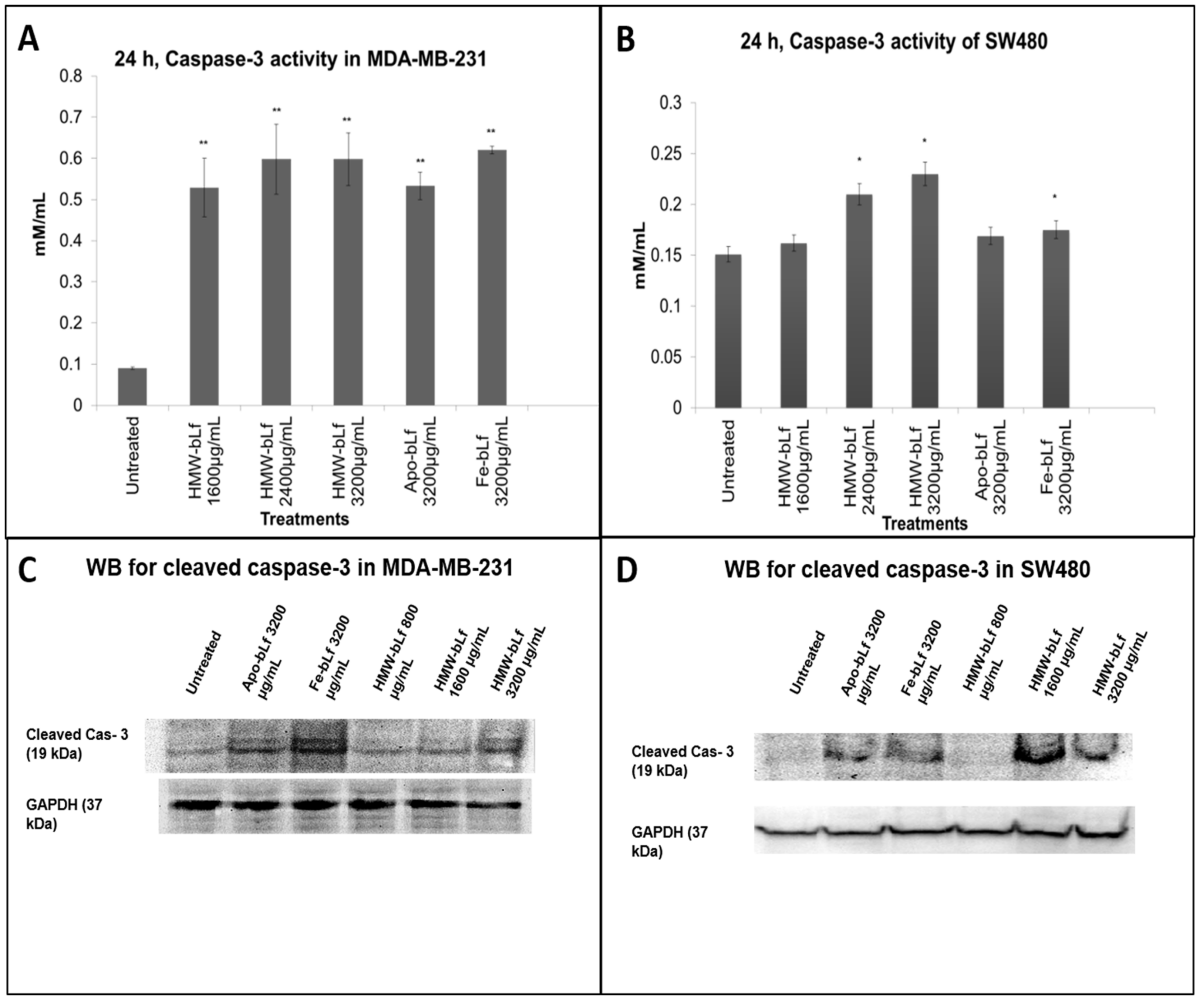
Caspase-3 activation. A and B represent the increased caspase-3 activity measurements upon treatment with HMW-bLf in MDA-MB-231 and SW480 cells, respectively. (* P<0.05 and ** P<0.01). Panels C and D are the respective Western blots showing an increase in cleaved caspase-3 expression upon treatments in MDA-MB-231 and SW480 cells.

This was further confirmed by studying the release of caspase-3, considered as the final executioner enzyme in the apoptotic pathway [45]. Treatment with HMW-bLf induced a statistically significant increase in the levels of caspase-3 secretion in both MDA-MB-231 and SW480 cells, thereby confirming the induction of cell death by apoptosis, (Figure 6 A and B). In both SW480 and MDA-MB-231 cells, HMW-bLf treatment significantly (*p<0.01*) up-regulated caspase-3 levels, and in SW480 cells the effect was also significant when compared with control Fe-bLf at 3200 µg mL^-1^. The rapid internalization of HMW-bLf into the cytoplasm and nuclei of cancer cells seems to lead to the initiation of gene transcription within the cell to trigger apoptotic signals thereby, resulting in cell death via apoptosis. We and other researchers have also shown that internalization of NM-bLf into the cell and nucleus can regulate gene transcription of its receptors, cytokines such as transforming growth factor-β and survivin [1], [46], [47]. bLf has been shown to activate both extrinsic and intrinsic apoptotic pathways through activation of different caspases [1], [7]. To confirm the results obtained using the caspase-3 activity assay, Western blot was performed for cleaved caspase-3, which is the active form of the apoptosis activator enzyme. Both SW480 and MDA-MB-231 cells show upregulation of the cleaved caspase-3 expression upon treatment with HMW-bLf. Especially, high expression of cleaved caspase-3 is seen in the 3200 µg mL^−1^ treatments of both Fe-bLf and HMW-bLf in MDA-MB-231 and with 1600 µg mL^−1^ in SW480 (Figure 6C and D). This indicates the ability HMW-bLf to induce apoptosis by activating caspase-3.

## Conclusions

In summary, our current *in vitro* study using breast and colon cancer cells showed for the first time the anticancer efficacy of ≥250 kDa HMW-biomacromolecular complex containing bLf. HMW-bLf was purified to homogeneity from Australian bovine colostrum. We have identified its unprecedented and interesting properties. HMW-bLf besides having molecular and structural similarities to Apo-bLf in terms of iron content also retains its antibody, and receptor binding properties. It possesses unique features such as higher thermal stability and better resistance against gut enzyme digestion than other forms of bLf monomer. Furthermore, HMW-bLf displayed stronger anti-cancer properties in terms of cytotoxicity and anti-cell proliferation activity. The possible actin degradation due to increased caspase-3 activity thereby, leading to apoptosis further signifies the need to explore the exact level of interesting interactions exhibited by HMW-bLf in modulating cancer cell death. The purified sample tested for its anticancer activities was obtained through final step of dialysis with 100 kDa MW cut off membrane, and thus devoid of any contamination with bLf monomer and other low molecular weight whey proteins such as lysozyme (14.6 kDa), α-lactalbumin -14.12(kDa), β-lactoglobulin – (22.40 kDa), αs_1_-casein – (33.30 kDa), β-casein – (37.50 kDa). The discovery of functionally bioactive HMW-bLf in this study has opened up greater scope for future research, considering the inherent multifunctional nature of bLf with its potential in improving human health. Through preclinical and clinical studies, we and others have shown that NM-bLf and Fe-bLf can not only inhibit tumor development but also reduce growth and metastasis of solid tumors [5], [6], [16]. Because of its widely reported multifunctional properties, and approval by FDA (US) and European Food Safety Authority as a dietary supplement in food products [38], [39] NM-bLf is gaining recent attention as an important therapeutic and nutraceutical against cancer, chronic inflammatory, viral and microbial diseases. In this regard, further studies are therefore, needed to decipher the structural and functional nature of HMW-bLf with more powerful techniques for its in-depth molecular organization and biophysical characterization. This will lead to the identification of similarities and differences in the activities displayed by these two forms of bLf, and help in understanding the true potential bLf as a multifunctional bio-macromolecule, in meeting the aims of modern medicine.

## Supporting Information

File S1 **Figures S1–S4.** Figure S1. Representative microscopy images showing trypan blue exclusion assay when the cells were grown in their respective growth media with 1% FBS for 24 h, indicating the >98% viability. This indicates that serum deprivation on incubation with bLf treatments for cellular uptake in 1% FBS containing assay media does not compromise the ability of the cells to exclude the dye and they remained healthy with intact membranes for cellular uptake of bLf. Magnifications 40X. Figure S2. Representative graph of the percentage iron content in the different forms of bLf. Figure S3. High resolution graph of FTIR spectra. The Fe-O vibration band appears at 560 cm-1 in the FTIR spectrum of Fe-bLf, and it is not pronounced in the other three spectra suggesting the high iron content in Fe-bLf and confirming iron content estimation. Figure S4. Cell death (mortality count) in FHs 74 Int as measured by Flow cytometry using propidium iodide staining (* p<0.05). Fe-bLf was used as a control. Figure S5: Confocal microscopy images showing FHs 74 Int cells (of normal intestinal origin) also take up HMW-bLf in a time dependent fashion. The internalized HMW-bLf was detected by indirect immunofluorescence using goat anti-bovine lactoferrin (Bethyl Laboratories) antibody at a dilution of 1∶200 in PBS at 37°C for 1 h. The primary antibody was then removed and after washing, cells were incubated with anti-goat IgG-FITC conjugate (Sigma-Aldrich) and counterstained for nucleus with DAPI (blue) in fluorshield (Sigma-Aldrich). Scale bar = 25 µm.
(DOCX)

## References

[1] Kanwar RK, Kanwar JR (2013) Immunomodulatory lactoferrin in the regulation of apoptosis modulatory proteins in cancer. Protein Pept Lett 20: 450–458. 23016584 10.2174/092986613805290417

[2] Baker EN, Baker HM (2005) Molecular structure, binding properties and dynamics of lactoferrin. Cell Mol Life Sci 62: 2531–2539. 16261257 10.1007/s00018-005-5368-9 PMC11139133

[3] Gutteridge JM, Paterson SK, Segal AW, Halliwell B (1981) Inhibition of lipid peroxidation by the iron-binding protein lactoferrin. Biochem J 199: 259. 7337708 10.1042/bj1990259 PMC1163360

[4] Tomita M, Wakabayashi H, Shin K, Yamauchi K, Yaeshima T, et al (2009) Twenty-five years of research on bovine lactoferrin applications. Biochimie 91: 52–57. 18585434 10.1016/j.biochi.2008.05.021

[5] Kanwar JR, Mahidhara G, Kanwar RK (2012) Novel alginate-enclosed chitosan-calcium phosphate-loaded iron-saturated bovine lactoferrin nanocarriers for oral delivery in colon cancer therapy. Nanomedicine 7: 1521–1550. 22734611 10.2217/nnm.12.29

[6] Tsuda H, Kozu T, Iinuma G, Ohashi Y, Saito Y, et al (2010) Cancer prevention by bovine lactoferrin: from animal studies to human trial. Biometals 23: 399–409. 20407806 10.1007/s10534-010-9331-3

[7] Gibbons JA, Kanwar RK, Kanwar JR (2011) Lactoferrin and cancer in different cancer models. Front biosci (Scholar edition) 3: 1080. 10.2741/212 21622257

[8] Stelwagen K, Carpenter E, Haigh B, Hodgkinson A, Wheeler T (2009) Immune components of bovine colostrum and milk. J Anim Sci 87: 3–9. 18952725 10.2527/jas.2008-1377

[9] Kanyshkova T, Buneva V, Nevinsky G (2001) Lactoferrin and its biological functions. Biochemistry (Moscow) 66: 1–7. 11240386 10.1023/a:1002817226110

[10] Mantel C, Miyazawa K, Broxmeyer HE (1994) Physical characteristics and polymerization during iron saturation of lactoferrin, a myelopoietic regulatory molecule with suppressor activity. Lactoferrin: Springer. pp. 121–132. 10.1007/978-1-4615-2548-6_12 7762423

[11] Nevinskii AG, Soboleva SE, Tuzikov FV, Buneva VN, Nevinsky GA (2009) DNA, oligosaccharides, and mononucleotides stimulate oligomerization of human lactoferrin. J Mol Recognit 22: 330–342. 19382280 10.1002/jmr.952

[12] Wang H, Hurley WL (1998) Identification of lactoferrin complexes in bovine mammary secretions during mammary gland involution. J Dairy Sci 81: 1896–1903. 9710757 10.3168/jds.S0022-0302(98)75761-3

[13] Brisson G, Britten M, Pouliot Y (2007) Heat-induced aggregation of bovine lactoferrin at neutral pH: Effect of iron saturation. Int Dairy J 17: 617–624.

[14] Kanwar JR, Kanwar RK (2009) Gut health immunomodulatory and anti-inflammatory functions of gut enzyme digested high protein micro-nutrient dietary supplement-Enprocal. BMC immunol 10: 7. 19183498 10.1186/1471-2172-10-7 PMC2667481

[15] Van Berkel P, Geerts M, Van Veen H, Kooiman P, Pieper F, et al (1995) Glycosylated and unglycosylated human lactoferrins both bind iron and show identical affinities towards human lysozyme and bacterial lipopolysaccharide, but differ in their susceptibilities towards tryptic proteolysis. Biochem J 312: 107–114. 7492299 10.1042/bj3120107 PMC1136233

[16] Kanwar JR, Palmano KP, Sun X, Kanwar RK, Gupta R, et al (2008) ‘Iron-saturated'lactoferrin is a potent natural adjuvant for augmenting cancer chemotherapy. Immunol cell biol 86: 277–288. 18268518 10.1038/sj.icb.7100163

[17] Fujie Y, Yamamoto H, Ngan CY, Takagi A, Hayashi T, et al (2005) Oxaliplatin, a potent inhibitor of survivin, enhances paclitaxel-induced apoptosis and mitotic catastrophe in colon cancer cells. JPN J Clin Oncol 35: 453–463. 16024531 10.1093/jjco/hyi130

[18] Sato K, Shiba M, Shigematsu A, Teduka N, Tomizawa A (2004) Method for producing lactoferrin. Google Patents.

[19] Harmon RJ, Schanbacher FL, Ferguson LC, Smith KL (1976) Changes in lactoferrin, immunoglobulin G, bovine serum albumin, and alpha-lactalbumin during acute experimental and natural coliform mastitis in cows. Infect Immun 13: 533–542. 770332 10.1128/iai.13.2.533-542.1976 PMC420644

[20] Castellino FJ, Fish WW, Mann KG (1970) Structural studies on bovine lactoferrin. J Biol Chem 245: 4269–4275. 5532223

[21] Persson BA, Lund M, Forsman J, Chatterton DE, Akesson T (2010) Molecular evidence of stereo-specific lactoferrin dimers in solution. Biophys Chem 151: 187–189. 20674143 10.1016/j.bpc.2010.06.005

[22] F.L. Schanbacher KLS, Ferguson LC (1971) The similarity of bovine lactoferrin dimer to IgG2. Fed Proc, 30: p. 532.

[23] Bennett RM, Bagby GC, Davis J (1981) Calcium-dependent polymerization of lactoferrin. Biochem biophys res comm 101: 88–95. 6793043 10.1016/s0006-291x(81)80014-9

[24] Klimeš J, Jagoš P, Bouda J, Gajdůšek S (1986) Basic qualitative parameters of cow colostrum and their dependence on season and post partum time. Acta Veterinaria Brno 55: 23–39.

[25] Sanchez L, Aranda P, Perez MD, Calvo M (1988) Concentration of lactoferrin and transferrin throughout lactation in cow's colostrum and milk. Biol Chem Hoppe Seyler 369: 1005–1008. 3228487 10.1515/bchm3.1988.369.2.1005

[26] Soboleva S, Tuzikov F, Tuzikova N, Buneva V, Nevinsky G (2009) DNA and oligosaccharides stimulate oligomerization of human milk lactoferrin. Mol Biol 43: 142–149. 19334538

[27] DEPT. OF HEALTH E, AND WELFARE PUBLIC HEALTH SERVICE FOOD AND DRUG ADMINISTRATION *ORA/ORO/DEIO/IB* (1985) Inspection Technical Guides, Bacterial Endotoxins/Pyrogens.

[28] Xavier PL, Chaudhari K, Verma PK, Pal SK, Pradeep T (2010) Luminescent quantum clusters of gold in transferrin family protein, lactoferrin exhibiting FRET. Nanoscale 2: 2769–2776. 20882247 10.1039/c0nr00377h

[29] Kussendrager K (1994) Effects of heat treatment on structure and iron-binding capacity of bovine lactoferrin. FIL-IDF. Secretariat general.

[30] Conesa C, Sánchez L, Pérez M-D, Calvo M (2007) A calorimetric study of thermal denaturation of recombinant human lactoferrin from rice. J Agr Food Chem 55: 4848–4853. 17503830 10.1021/jf063335u

[31] Rüegg M, Moor U, Blanc B (1977) A calorimetric study of the thermal denaturation of whey proteins in simulated milk ultrafiltrate. J Dairy Res 44: 509–520.

[32] Baker HM, Baker EN (2004) Lactoferrin and iron: structural and dynamic aspects of binding and release. Biometals 17: 209–216. 15222467 10.1023/b:biom.0000027694.40260.70

[33] Troost FJ, Steijns J, Saris WH, Brummer RJ (2001) Gastric digestion of bovine lactoferrin in vivo in adults. J Nutr 131: 2101–2104. 11481401 10.1093/jn/131.8.2101

[34] Sitaram MP, McAbee DD (1997) Isolated rat hepatocytes differentially bind and internalize bovine lactoferrin N- and C-lobes. Biochem J 323 (Pt 3): 815–822. 10.1042/bj3230815 PMC1218387 9169617

[35] Roseanu A, Florian PE, Moisei M, Sima LE, Evans RW, et al (2010) Liposomalization of lactoferrin enhanced its anti-tumoral effects on melanoma cells. Biometals 23: 485–492. 20191307 10.1007/s10534-010-9312-6

[36] Duarte D, Nicolau A, Teixeira J, Rodrigues L (2011) The effect of bovine milk lactoferrin on human breast cancer cell lines. J Dairy Sci 94: 66–76. 21183018 10.3168/jds.2010-3629

[37] Iigo M, Kuhara T, Ushida Y, Sekine K, Moore MA, et al (1999) Inhibitory effects of bovine lactoferrin on colon carcinoma 26 lung metastasis in mice. Clin Exp Metastasis 17: 35–40. 10390145 10.1023/a:1026452110786

[38] Rulis AM (2001) Agency Response Letter GRAS Notice No. GRN 000077.

[39] EFSA Panel on Dietetic Products NaAN (2012) Scientific Opinion on bovine lactoferrin. EFSA Journal 10(7):2811 [14 pp.].

[40] Smith SM, Wunder MB, Norris DA, Shellman YG (2011) A Simple Protocol for Using a LDH-Based Cytotoxicity Assay to Assess the Effects of Death and Growth Inhibition at the Same Time. PLoS ONE 6: e26908. 22125603 10.1371/journal.pone.0026908 PMC3219643

[41] Lonnerdal B, Jiang R, Du X (2011) Bovine lactoferrin can be taken up by the human intestinal lactoferrin receptor and exert bioactivities. J Pediatr Gastroenterol Nutr 53: 606–614. 21832946 10.1097/MPG.0b013e318230a419

[42] Samarasinghe RM, Kanwar RK, Kanwar JR (2014) The effect of oral administration of iron saturated-bovine lactoferrin encapsulated chitosan-nanocarriers on osteoarthritis. Biomaterials 35: 7522–7534. 24933511 10.1016/j.biomaterials.2014.04.109

[43] Kanwar JR, Mahidhara G, Roy K, Sasidharan S, Krishnakumar S, et al.. (2014) Fe-bLf nanoformulation targets survivin to kill colon cancer stem cells and maintains absorption of iron, calcium and zinc. Nanomedicine: 1–21. 10.2217/nnm.14.132 25017148

[44] Kothakota S, Azuma T, Reinhard C, Klippel A, Tang J, et al (1997) Caspase-3–generated fragment of gelsolin: effector of morphological change in apoptosis. Science 278: 294–298. 9323209 10.1126/science.278.5336.294

[45] Wolf BB, Schuler M, Echeverri F, Green DR (1999) Caspase-3 is the primary activator of apoptotic DNA fragmentation via DNA fragmentation factor-45/inhibitor of caspase-activated DNase inactivation. J Biol Chem 274: 30651–30656. 10521451 10.1074/jbc.274.43.30651

[46] Fleet JC (1995) A New Role for Lactoferrin: DNA Binding and Transcription Activation. Nutrition Reviews 53: 226–227. 7501307 10.1111/j.1753-4887.1995.tb01556.x

[47] Jiang R, Lonnerdal B (2011) Apo-lactoferrin regulates transcription of the TGF beta 1 gene and may thus stimulate intestinal differentiation. The FASEB Journal 25: 340.346.

