# Supplementary material for: Identification of Unprecedented Anticancer Properties of High Molecular Weight Biomacromolecular Complex Containing Bovine Lactoferrin (HMW-bLf)
Source: PLoS One. 2014 Sep 15;9(9):e106568. doi: 10.1371/journal.pone.0106568 (PMC4164354; doi:10.1371/journal.pone.0106568)
Supplement: File S1 — Figures S1–S4. Figure S1. Representative microscopy images showing trypan blue exclusion assay when the cells were grown in their respective growth media with 1% FBS for 24 h, indicating the >98% viability. This indicates that serum deprivation on incubation with bLf treatments for cellular uptake in 1% FBS containing assay media does not compromise the ability of the cells to exclude the dye and they remained healthy with intact membranes for cellular uptake of bLf. Magnifications 40X. Figure S2. Representative graph of the percentage iron content in the different forms of bLf. Figure S3. High resolution graph of FTIR spectra. The Fe-O vibration band appears at 560 cm-1 in the FTIR spectrum of Fe-bLf, and it is not pronounced in the other three spectra suggesting the high iron content in Fe-bLf and confirming iron content estimation. Figure S4. Cell death (mortality count) in FHs 74 Int as measured by Flow cytometry using propidium iodide staining (* p<0.05). Fe-bLf was used as a control. Figure S5: Confocal microscopy images showing FHs 74 Int cells (of normal intestinal origin) also take up HMW-bLf in a time dependent fashion. The internalized HMW-bLf was detected by indirect immunofluorescence using goat anti-bovine lactoferrin (Bethyl Laboratories) antibody at a dilution of 1∶200 in PBS at 37°C for 1 h. The primary antibody was then removed and after washing, cells were incubated with anti-goat IgG-FITC conjugate (Sigma-Aldrich) and counterstained for nucleus with DAPI (blue) in fluorshield (Sigma-Aldrich). Scale bar = 25 µm. (DOCX) [file pone.0106568.s001.docx]

**Identification of Unprecedented Anticancer Properties of High Molecular Weight Biomacromolecular Complex Containing Bovine Lactoferrin (HMW-bLf)**

*Fawzi Ebrahim^1^*†**, Jayanth Suryanarayanan Shankaranarayanan^1^*, Jagat R*. *Kanwar^1^, Sneha Gurudevan^1^, Uma Maheswari Krishnan^2^, Rupinder K*. *Kanwar^1^**

**Supplementary Information**

**Figure S1.**

**Figure S2.**

**Standard**

**Figure S3.**

**Figure S4.**

**Figure S5.**
